# A Strategy for Nonmigrating Plasticized PVC Modified with Mannich base of Waste Cooking Oil Methyl Ester

**DOI:** 10.1038/s41598-018-19958-y

**Published:** 2018-01-25

**Authors:** Puyou Jia, Meng Zhang, Lihong Hu, Fei Song, Guodong Feng, Yonghong Zhou

**Affiliations:** 1 Institute of Chemical Industry of Forest Products, Chinese Academy of Forestry (CAF), Jiangsu Province, No. 16 Suojin North Road, Nanjing, 210042 P.R. China; 2 National Engineering Lab for Biomass Chemical Utilization, Jiangsu Province, No. 16 Suojin North Road, Nanjing, 210042 P.R. China; 3 Key Lab on Forest Chemical Engineering, State Forestry Administration, Jiangsu Province, No. 16 Suojin North Road, Nanjing, 210042 P.R. China; 4 Key Lab of Biomass Energy and Materials, Jiangsu Province, No. 16 Suojin North Road, Nanjing, 210042 P.R. China

## Abstract

The waste cooking oil (WCO) production from the catering industry and food processing industry causes serious environmental, economic and social problems. However, WCO can be used for the preparation of fine chemicals such as internal plasticizer. With this aim, this work is focused on preparing internal plasticizer by using WCO and determining technical viability of non-migration poly (vinyl chloride) (PVC) materials. The mannich base of waste cooking oil methyl ester (WCOME) was synthesized from WCO via esterification, interesterification and mannich reaction, which was used to produce self-plasticization PVC materials as an internal plasticizer. The results showed that the PVC was plasticized effectively. Self-plasticization PVC films showed no migration in n-hexane, but 15.7% of dioctyl phthalate (DOP) leached from DOP/PVC(50/50) system into n-hexane. These findings transformed the traditional plastic processing technology and obtained cleaner production of no migration plasticizer from WCO.

## Introduction

Waste cooking oil (WCO) is an oil-based substance, which has been used to make foods, but no longer suitable for eating. WCO is mainly generated from these urban areas with high density population and high consumption of refined vegetable oils. WCO production from the catering industry and food processing industry causes serious environmental, economic and social problems^1,2^. However, it can be used and transformed into value-added products such as biodiesel^3,4^. The unique composition of WCO is triglyceride and fatty acid, which can be transformed into biodiesel production via conventional transterification method^5,6^. The strategy for transterification of triglyceride and fatty acid has been widely investigated^7,8^. It has been well-reported that WCO acted very well as alternative to petrochemical resources for biodiesel production^9^. The strategy of biodiesel production from WCO with low price about internally plasticized PVC materials from WCO is one of the best ways to utilize it efficiently and economically.

Plasticizer is an important polymer additive, which has been widely used in plastics, rubbers, adhesives, cellulose and so on. The common used plasticizers are phthalate esters, accounting for 70% of the global plasticizer demand in 2014^10^. However, the phthalate esters are easy to migrate from polymer matrix during processing and using with increasing time, which decreases the service life of polymer products, as well as potential toxicity to human body^11,12^. Recently, epoxidized vegetable oil^13,14^, polymer plasticizer^13,15^, polyol ester^16,17^ and phosphate plasticizer^14^ have been reported as alternative plasticizers, which suppresses the migration from PVC products in a certain degree, but epoxidized vegetable oil, polyol ester and phosphate plasticizer will migrate from PVC products with increasing time. The effective strategy to avoid the migration of plasticizers is covalent attachment of the plasticizer onto the PVC backbone^18–20^.

The production of internally plasticized PVC materials can transform the traditional plastic processing technology, which mainly includs hot mixing, dry mixing and solvent casting method. The hot mixing and dry mixing with high energy consumption and undermixing, and solvent casting method with a amount of organic reagents restricts their further application. The internally plasticized PVC materials can be directly produced plastic products without further plasticization process, which will spur a revolution in plastic processing technology. In this study, mannich base of waste cooking oil methyl ester (WCOME) was synthesized from WCO, the chemical structure was detected using Fourier transform infrared spectroscopy (FT-IR) and Hydrogen nuclear magnetic resonance spectroscopy (^1^H NMR), which was used as a novel non-migration plasticizer for self-plasticization PVC materials. The structures of self-plasticization PVC materials were investigated with FT-IR, ^1^H NMR and gel permeation chromatography (GPC). The properties of the PVC materials such as Tg, thermal stability, migration resistance and mechanical properties were also investigated. These properties of self-plasticization PVC were compared with neat PVC and PVC/DOP system, and the internal plasticization mechanism was also discussed. The modified PVC materials were expected to be commercial application in producing these products with high requirement in migration resistance such as food packing, toys and medical devices. The technical viability of non-migration PVC was determined, and the strategy was expected to improving the traditional plastic processing technology.

## Results

### Chemical structure of mannich base of WCOME and reaction mechanism

**Figure 1:**
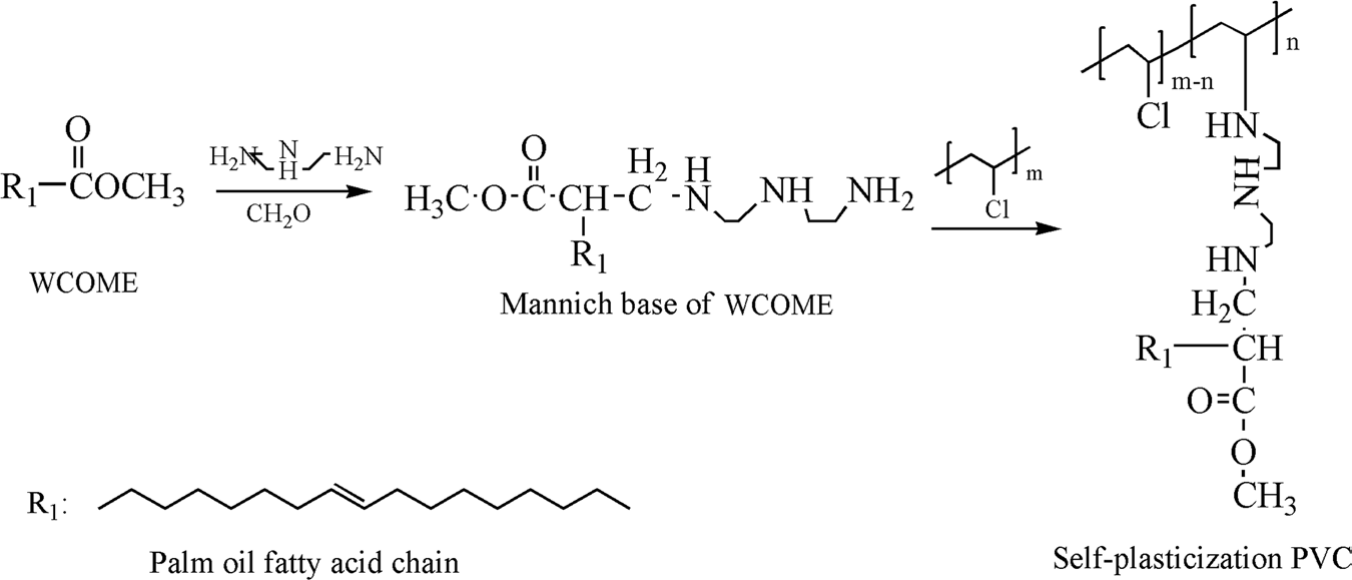
Preparation of self-plasticization PVC.

**Figure 2:**
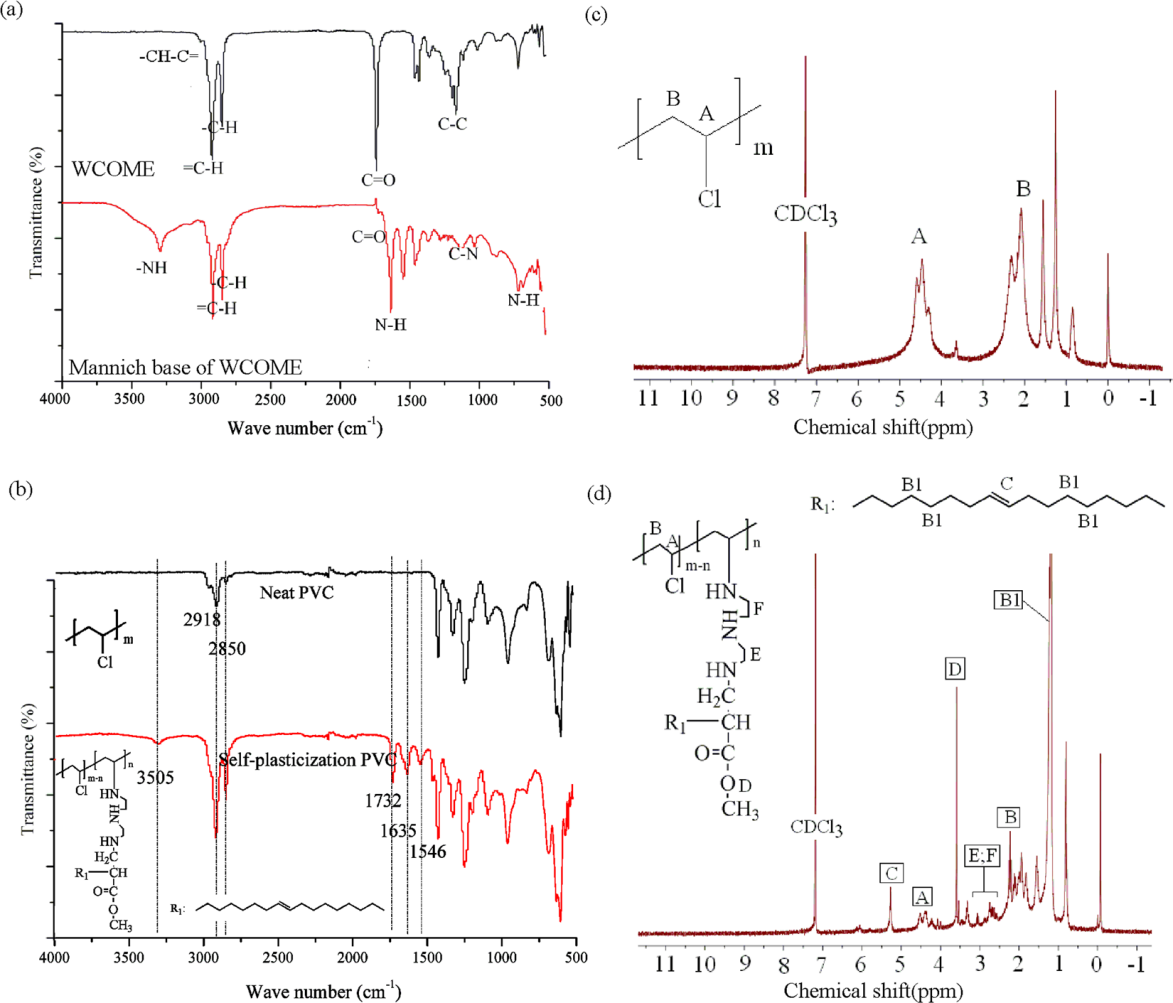
(**a**) FT-IR spectra of WCOME and mannich base of WCOME; (**b**) FT-IR spectra of neat PVC and self-plasticization PVC; (**c**) ^1^H NMR spectra of neat PVC; (**d**) ^1^H NMR spectra of self-plasticization PVC.

Figure 1 shows the synthetic route of mannich base of WCOME. Mannich reaction has bright prospects for organic synthesis. Many compounds with structural complexity and diversity can be obtained via Mannich reaction from simple and readily available starting materials without separation of intermediates. The FT-IR spectra of WCOME and mannich base of WCOME were monitored and presented in Fig. 2(a). As seen from the FT-IR spectrum of WCOME, the strong absorption peaks appeared at 3006, 2921, 2852, 1741, 1169 cm^−1^, which were attributed to CH-C=, =C-H, -C-H, C=O and C-C bonds, respectively^21^. In the FT-IR spectrum of mannich base of WCOME, the disappearance of the C=O characteristic absorption peak with concomitant appearance of the -NH at 3296 cm^−1^, and the absorption peak at 1636 cm^−1^ which represents the rocking vibration of N-H, the absorption peak at 1035 cm^−1^ corresponds to the vibration of C-N, the weak and broad peaks at 686 cm^−1^ were attributed to N-H twisting vibrations^22,23^, which is indicative of the formation of the target products.

**Figure 3:**
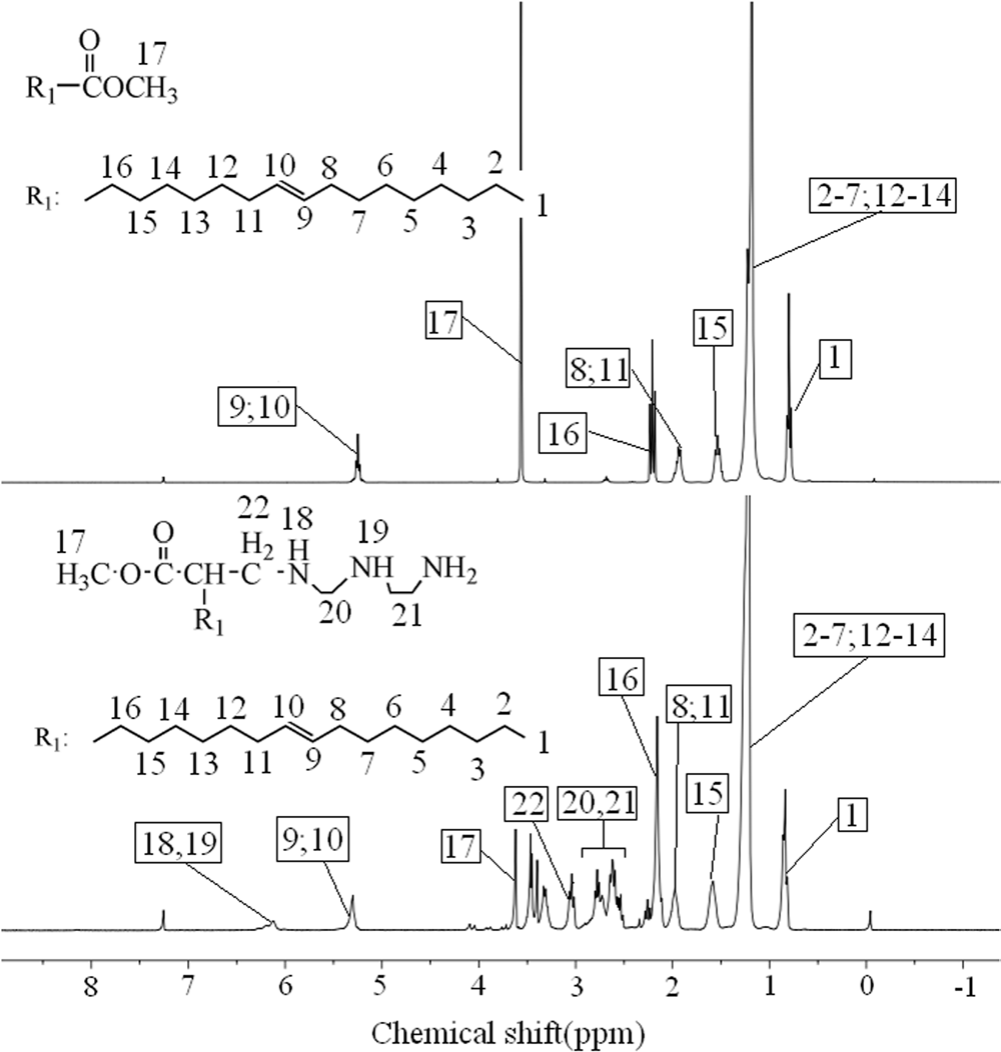
^1^H NMR spectra of WCOME and mannich base of WCOME.

The chemical structure of WCOME and mannich base of WCOME were also confirmed by ^1^H NMR, all the peaks in the ^1^H NMR spectrum are easily assigned in Fig. 3, as shown in the ^1^H NMR spectrum of WCOME, the peak at 0.8 ppm was attributed the protons of methyl groups on the unsaturated long fatty acid chains. The strong peak at 1.2 ppm was assigned to the protons of methylene groups. The groups appeared at 1.5, 1.9 and 2.2 ppm corresponds to the other methylene groups. The strong signals appeared at 3.5 ppm was originated from the methyl groups connected to ester groups. The signals at 5.2 ppm were attributed to the protons of olefin groups^24^.In the ^1^H NMR spectrum of mannich base of WCOME, the appearance of new peaks at 2.6 and 2.7 ppm were assigned to the methylene groups derived from diethylenetriamine, and the new peaks appeared at around 6.1 ppm was corresponded to protons of amine groups^22^, which confirmed the suggested structure of the mannich base.

**Figure 4:**
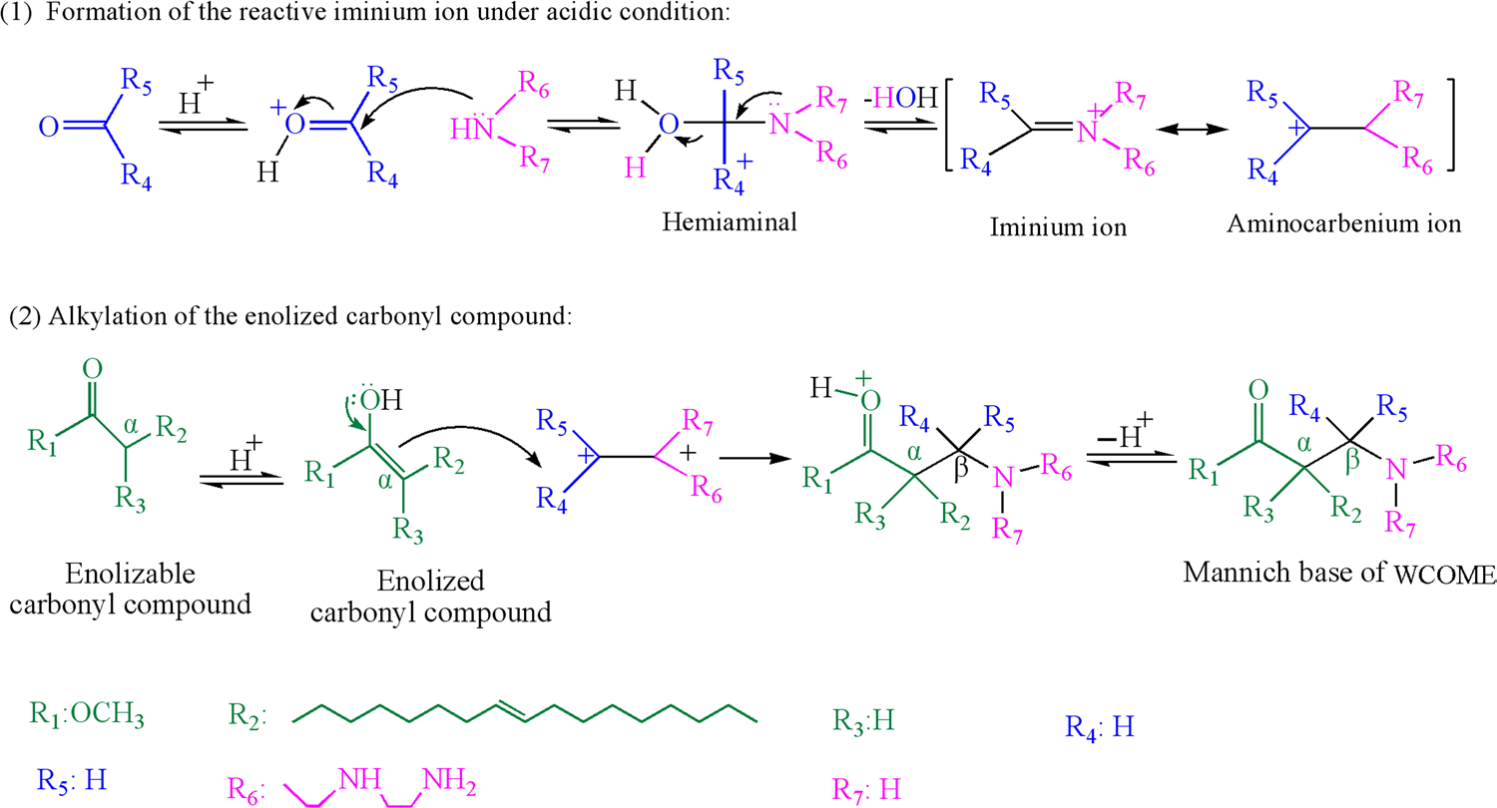
Synthesis mechanism of mannich base of WCOME.

The synthesis mechanism of mannich base of WCOME was discussed according to the related literatures^25–28^. The synthesis included two stages. As seen from Fig. 4, the first stage was formation of the reactive iminium ion under acidic condition. In this stage, carbonyl groups of WCOME were protonation in sulfuric acid solution, then nucleophilic addition occured between the diethylenetriamine and protonated carbonyl groups. Aminocarbenium ion was obtained after electron transfer of nitrogen. The second stage was alkylation of the enolized carbonyl compound. Enolized carbonyl compound was gotten under acidic condition in the process. Enol structure with active hydrogen was attacked by the iminium ion as nucleophile. Finally, mannich base of WCOME was obtained after losing protons.

### Chemical structures of neat PVC and self-plasticization PVC

Chemical structures of neat PVC and self-plasticization PVC were detected using FT-IR and ^1^H NMR. As seen from the Fig. 2(b), these peaks at 3505, 2918, 2850, 1732 and 1635 cm^−1^, which are assigned to the -NH stretching vibrations, C-H(sp^3^), C-H(sp^2^), C=O and N-H rocking vibration respectively^21^, were increased gradually with more displacement of chlorine with mannich base of WCOME, indicating it was connected to the chemical structure of self-plasticization PVC successfully.

The ^1^H NMR spectra of neat PVC and self-plasticization PVC materials were showed in Fig. 2(c and d), respectively. As seen from Fig. 2(c), the peak at 4.45 ppm was attributed to protons of CH-Cl (peak A of ^1^H NMR spectra of PVC), and the peak at 2.06 ppm was assigned to protons of CH_2_ (peak B of ^1^H NMR spectra of PVC)^29,30^. New peak appeared in the ^1^H NMR spectra of self-plasticization PVC with more displacement of chlorine with mannich base of WCOME compared with ^1^H NMR spectra of neat PVC. As seen from the Fig. 2(d), it is clearly to observe that the signals at 1.25, 2.80, 3.58 and 5.29 ppm were attributed to the protons of -CH_2_-, O-CH_3_, -NH-CH_2_-CH_2_-NH- and -CH=CH-, respectively^31^, which appeared stronger with more displacement of chlorine with mannich base of WCOME, but the signal at 4.5 ppm corresponds to the protons of CH-Cl presented weaker gradually, which indicated that the self-plasticization PVC was obtained.

**Figure 5:**
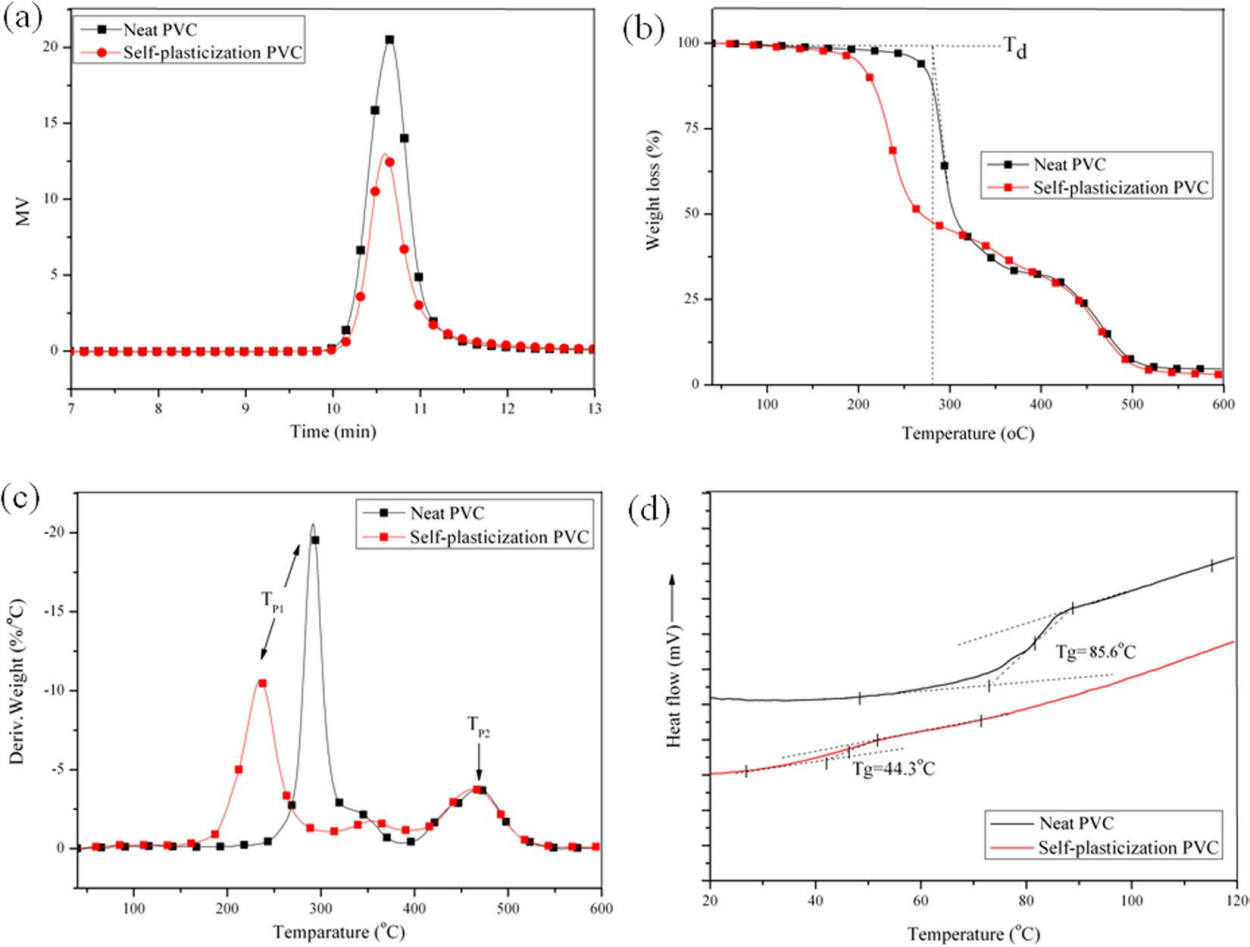
(**a**) GPC spectra of neat PVC and self-plasticization PVC; (**b**) TGA curves of neat PVC and self-plasticization PVC; (**c**) DTG curves of neat PVC and self-plasticization PVC; (**d**) DSC curves of neat PVC and self-plasticization PVC.

GPC analysis can be used as a method to evaluate the reaction level by examining the molar mass change^32,33^. GPC spectra of neat PVC and self-plasticization PVC were shown in Fig. 5(a), and data of number average molar mass (*M*_n_), weight-average molar mass (*M*_W_), Z-average molar mass(*M*_Z_) and dispersity were summarized in Table 1. *M*_n_, *M*_W_ and *M*_Z_ of PVC materials increased gradually from 15100, 18900 and 23000 g/mol to 19700, 24400 and 30600 g/mol, respectively. In addition, GPC peak of self-plasticization PVC showed a clear shift to higher molar mass region comparing with that of neat PVC, indicating that chlorine atoms of PVC were substituted with mannich base of WCOME. Self-plasticization PVC presented a single GPC peak with a clear shift to a higher molar mass region, which indicated that there was no homopolymer contamination of coupling reactions. Peak area of self-plasticization PVC decreased with increasing substituting of chlorine atoms with mannich base of WCOME can be found in Fig. 5(a), the decrease in peak area was attributed to filtration of highly branched self-plasticization PVC by organic membrane during dissolution process with chromatographic purity as solvent.

**Table 1:** Relative molecular mass and distribution of PVC materials.

Sample	Mn (g/mol)	Mw (g/mol)	Mz (g/mol)	Dispersity
Neat PVC	15100	18900	23000	1.2
self-plasticization PVC	19700	24400	30600	1.2

Mn: Number average molar mass.

Mw: Weight-average molar mass.

Mz: Z-average molar mass.

Thermal properties and plasticity of neat PVC and self-plasticization PVC. TGA and derivative thermogravimetric analysis(DTG) curves obtained for neat PVC and self-plasticization PVC were showed in Fig. 5(b and c), respectively. Table 2 summarized the thermal data including Tg, thermal degradation temperature (T_d_) and peak temperature of thermal degradation (T_p1_ and T_p2_). Neat PVC and self-plasticization PVC showed two thermal degradation stages, which was assigned to dehydrochlorination of PVC and cyclization of conjugated polyene sequences^34^. The characteristic temperatures of the T_d_, T_p1_ and T_p2_ for neat PVC decreased gradually from 278.4, 291.5 and 467.4 °C to 210.7, 234.3 and 463.7 °C with increasing substitution of chlorine atoms with mannich base of WCOME, which illustrated that self-plasticization PVC was less thermal stable than neat PVC, because many active secondary amine groups existed in the structure of self-plasticization PVC.

**Table 2:** Thermal properties of PVC materials.

Sample	T_d_ (°C)	T_p1_ (°C)	T_p2_ (°C)	T_g_ (°C)
Neat PVC	278.4	291.5	467.4	85.6
Self-plasticization PVC	210.7	234.3	463.1	44.3

T_d_: Thermal degradation temperature.

T_p1_: Peak 1 temperature of thermal degradation.

T_g_: Glass transition temperature.

T_p2_: Peak 2 temperature of thermal degradation.

Neat PVC with stiff backbone has high glass transition temperature(Tg) at around 90 °C. The addition of plasticizer into the PVC matrix will increase distance of PVC chains and make macromolecule structure polymer easy to move, increasing free volume of polymer and reducing Tg. The substituting of chlorine atoms with mannich base of WCOME will increase distance between PVC chains and reduce intermolecular force. An internal plasticization will be expected to reducing Tg of PVC. The DSC curves were showed in Fig. 5(d), which can be found that a single change in heat capacity of Tg could be observed in the DSC curve of self-plasticization PVC, illustrating the reaction of PVC and mannich base of WCOME was completed, and there isn’t any free mannich base of WCOME existed in the polymer. Tg of self-plasticization PVC decreased from 85.6 °C to 44.3 °C, when chlorine atoms in PVC was substituted with mannich base of WCOME, suggesting the internal plasticized effect on PVC was played.

The mechanical properties of PVC materials were tested. As seen from Table 3, Tensile strength decreased from 30.63 to 21.36 MPa with increasing substitution of chlorine atom with mannich base of WCOME, and the elongation at break increased from 163.03% to 330.92%, indicating the PVC was plasticized effectively. It suggested that substitution of chlorine atom with mannich base of WCOME increased the distance of PVC chains and decreased the interaction force between the macromolecule, and then increased the lubricating property of PVC chains. It caused the ordered structure of PVC chains was destroyed, and made the PVC materials present more flexible than neat PVC films.

**Table 3:** Tensile data of PVC materials.

Sample	Tensile strength (MPa)	Elongation at break (%)	Modulus of elasticity (MPa)
Neat PVC	30.63 ± 0.87	163.03 ± 5.41	203.79 ± 3.25
Self-plasticization PVC	21.36 ± 0.35	330.92 ± 6.30	80.75 ± 1.26

Finally, we estimated the migration resistance of 50 wt.% DOP/PVC system and self-plasticization PVC film in n-hexane at 50 °C for 2 h. The results indicated that self-plasticization PVC film showed no migration in n-hexane, but 15.7% of DOP leaching from DOP/PVC system to n-hexane. The zero migration property can maintain physical and chemical properties of PVC products long-time stable.

## Conclusions

In this work, WCO was used and transformed into mannich base of WCOME, which was used to synthesis self-plasticization PVC material as internal plasticizer. Chemical structure and properties of mannich base of WCOME and self-plasticization PVC were characterized. The results showed that *M*_n_, *M*_W_ and *M*_Z_ of self-plasticization PVC increased gradually from 15100, 18900 and 23000 g/mol to 19700, 24400 and 30600 g/mol with displacement of chlorine. Tg of self-plasticization PVC decreased from 85.6 °C to 44.3 °C. Tensile strength decreased from 30.63 MPa to 21.36 MPa, and the elongation at break increased from 163.03% to 330.92%, indicating the PVC was plasticized effectively. The Self-plasticization PVC film showed no migration in n-hexane, but 15.7% of DOP leached from DOP/PVC system into n-hexane. However, self-plasticization PVC was less thermal stable than neat PVC, because many active secondary amine groups existed in the structure of self-plasticization PVC material. This study provided a better way to enhance the added value of WCO. These findings are conducive to resolve the serious environment, economic and social problems caused by a large amount of WCO production from catering industry and food processing industry. The production of internally plasticized PVC materials can transform the traditional plastic processing technology. In addition, the internally plasticized PVC materials can be directly produced plastic products without further plasticization process, which will spur a revolution in plastic processing technology.

## Methods

### Materials

Phosphoric acid, methanol, sulphuric acid, hydrochloric acid, sodium bicarbonate, sodium chloride, sodium hydroxide, formaldehyde solution (37 wt.%), dioctyl phthalate (DOP), diethylenetriamine, dimethylformamide, clay were kindly provided by Nanjing Chemical Reagent Co., Ltd. All of these chemicals and reagents were analytical grade and used without further purification. Neat PVC was supplied by Hanwha (KM-31, South Korea). WCO was provided from Military and Civilian oil processing plants (Hailing district, Taizhou city, WCO was derived from palm oil after repeated use. The main components are palm oil, palmitoleic acid and solid impurities. Iodine value, 44 g I_2_/100 g). Acid value 127 mg KOH/g. Density 945 kg/m^3^(20 °C). Kinematic viscosity 4.2 mm^2^/s (40 °C). Impurities 1.35%.

### Pretreatment of WCO

WCO (1000 g) was decolorized with 60 g clay at 50 °C for 2 hour, and then filtered. 20 g of phosphoric acid solution (10 wt.%) was added to degelatinize at 50 °C for 30 min. The pretreatment of WCO was finished after washing with distilled water and vacuum distillation. The yield of WCO was 76.57%.

### Esterification of WCO

Pretreatment of WCO (100 g), methanol (30 g) and concentrated sulfuric acid (1.5 g) were mixed in a three-necked round-bottom which was equipped with a mechanical stirred, condenser pipe and thermometer. The mixture was stirred at 75 °C for 3 h to finish the esterification. Then the product was washed with 3 wt.% of saturated sodium bicarbonate solution and saturated sodium chloride solution, respectively. The esterification product was attained after vacuum distillation. (Yield 95.6%. Iodine value 53 g I_2_/100 g. Acid value 0.3 mg KOH/g. Density 886 kg/m^3^ at 20 °C. Kinematic viscosity 3.8 mm^2^/s at 40 °C).

### Synthesis of WCOME

To a stirred solution of esterification of WCO (100 g), methanol (40 g) and sodium hydroxide (2 g) in a three-necked round-bottom which was equipped with a mechanical stirred, condenser pipe and thermometer at 65 °C for 2 h. Then the mixture was washed with sulfuric acid solution and saturated sodium chloride solution. The mixture was settled for a while and layered. Then the upper WCOME layer was purified by vacuum distillation to remove water. The yield of WCOME was 96.3%.

### Synthesis of mannich base of WCOME

To a stirred solution of WCOME (27 g) and diethylenetriamine (10.3 g) was added 8.1 g of formaldehyde (37 wt.%) and 10 mL of hydrochloric acid solution in a three-necked round-bottom which was equipped with a mechanical stirred, condenser pipe and thermometer. The formaldehyde was dropped in the solution in 20 min. The mixture was stirred at 90 °C for 4 h. The resulting product mannich base of WCOME was The the product was obtained after removal of water.The yield of mannich base of WCOME was 94.1%.

### Synthesis of self-plasticization PVC

To a stirred solution of PVC (5 g) and mannich base of WCOME (4 g) in 80 mL of DMF was added in a round-bottom. The mixture was stirred at 80 °C for 2 h. Then the self-plasticization PVC was obtained after washing with 10 wt.% aqueous methanol solution and drying in an electrothermal blowing dry box at 60 °C.

### Preparation of PVC films

Self-plasticization PVC (3 g) was dissolved in 60 mL of THF. The mixture was stirred at 40 °C for 20 min until the solution presented transparent. Then the solution was poured into a glass petri dish (12 cm diameter) and dried in a drying box at 60 °C for 24 h to completely remove residual THF. The PVC film with a thickness of approximately 0.20 mm was obtained. Neat PVC film and 50 wt.% DOP/PVC film were obtained using the same method.

### Characterizations

Iodine value of WCOME was investigated according on ISO 3961-2009. Acid value of WCOME was tested according on NF EN ISO 660-1999. Kinematic viscosity was detected following ISO 6321-2002. Density of the waste cooking oil methyl ester was investigated following the Chinese standard GB/T 5518-2008. Fourier transform infrared spectrometry (FT-IR) was recorded on a Nicolet iS10 FT-IR (Nicolet Instrument Corporation, USA) fourier transformed infrared spectrophotometer. The spectra were acquired in the range of 4000 cm^−1^ to 500 cm^−1^ at a resolution of 4 cm^−1^. Peaks in the spectra were labeled automatically using OMNIC software (Thermo Electron Corporation, USA). ^1^H Nuclear Magnetic Resonance(NMR) of intermediate products and self-plasticization PVC were performed on an AV-300 NMR spectrometer (Bruker Instrument Corporation, Germany) at a frequency of 400 MHz. The process was carried out using CDCl_3_ as solvent and tetrametnylsilane (TMS) as an internal standard. The NMR data was processed using MestReNova software (Santiago de Compostela, Spain). The average molar mass and dispersity of neat PVC and self-plasticization PVC materials were investigated on a Gel Permeation Chromatography (GPC) measurement (Waters, USA) at 30 °C (flow rate: 1 mL/min, column: mixed PL gel 300 × 718 mm, 25 μm) using HPLC-grade THF as solvent. Neat PVC and self-plasticization PVC were brought into THF solution with concentration of 1–5 mg/mL. The thermogravimetric analysis (TGA) tests were carried out using a TG209F1 TGA thermal analysis instruments (Netzsch Instrument Corporation, Germany) in N_2_ atmosphere (50 mL/min) at a heating rate of 10 °C/min. The data was collected while the oven temperature was ranging from 40 °C to 600 °C. Differential scanning calorimeter (DSC) measurement was carried out under N_2_ atmosphere using a NETZSCH DSC 200 PC analyzer (Netzsch Instrument Corporation, Germany). The temperature was over a range of −40–120 °C at a heating of 20 °C/min. 5–10 mg neat PVC and self-plasticization PVC were weight and sealed in a 40 uL aluminum crucible, and immediately detected using DSC measurement. The DSC data was collected from first cycle of heating. The mechanical properties including tensile modulus, tensile strength, and elongation at break were investigated according ISO 527-1-2012 at 25 °C by using an E43.104 Universal Testing Machine (MTS Instrument Corporation, China) at a strain rate of 20 mm/min. All the datas was was collected using average value of five parallel tests. The leaching tests were performed were carried out according on ASTM D5227. Neat PVC and self-plasticization PVC films after weighting were immersed in n-hexane at 50 °C for 2 h. These PVC films were dried and reweighed. The extraction loss was calculated according to the Equation (1) and taken average value of five experiments.

1 $${\rm{Degree}}\,{\rm{of}}\,{\rm{migration}}=[({W}_{1}-{W}_{2})/{W}_{1})]\times 100$$

*W*_1_ = initial weight of test films, and *W*_2_ = final weight of test PVC films.
